# Association of physical activity with lung function in lung-healthy German adults: results from the KORA FF4 study

**DOI:** 10.1186/s12890-017-0562-8

**Published:** 2017-12-28

**Authors:** Agnes Luzak, Stefan Karrasch, Barbara Thorand, Dennis Nowak, Rolf Holle, Annette Peters, Holger Schulz

**Affiliations:** 1 Institute of Epidemiology I, Helmholtz Zentrum München - German Research Center for Environmental Health, Ingolstädter Landstr. 1, 85764 Neuherberg, Germany; 2 Institute and Outpatient Clinic for Occupational, Social and Environmental Medicine, University Hospital of Munich (LMU), Ziemssenstr. 1, 80336 Munich, Germany; 3 Institute of Epidemiology II, Helmholtz Zentrum München - German Research Center for Environmental Health, Ingolstädter Landstr. 1, 85764 Neuherberg, Germany; 4 Comprehensive Pneumology Center Munich (CPC-M), Member of the German Center for Lung Research, Max-Lebsche-Platz 31, 81377 Munich, Germany; 5 Institute of Health Economics and Health Care Management, Helmholtz Zentrum München - German Research Center for Environmental Health, Ingolstädter Landstr. 1, 85764 Neuherberg, Germany

**Keywords:** Lung function, Spirometry, Activity behavior, Accelerometer

## Abstract

**Background:**

In lung disease, physical activity (PA) yields beneficial health effects, but its association with the function of healthy lungs has rarely been studied. We investigated the association of accelerometer-based PA with spirometric indices, maximal inspiratory mouth pressure (PI_max_) and lung diffusion capacity in lung-healthy adults.

**Methods:**

In total, 341 apparently lung-healthy participants from the population-based KORA (Cooperative Health Research in the Region of Augsburg) FF4 cohort study (45% male, aged 48-68 years, 47% never smokers) completed lung function testing and wore ActiGraph accelerometers over a one week period at the hip. In adjusted regression analyses, moderate to vigorous PA (MVPA) was characterized as: sex-specific activity quartiles, achieving ≥ 10 consecutive minutes (yes vs. no), and meeting the WHO PA recommendations (yes vs. no).

**Results:**

Positive associations of MVPA-quartiles with forced expiratory volume in 1 s (FEV_1_), forced vital capacity (FVC), and corresponding Global Lung Function Initiative z-scores were found. Subjects in the most active quartile (> 47 or > 50 min/day for females and males, respectively) had 142 ml [95% CI: 23, 260] higher FEV_1_ and 155 ml [95% CI: 10, 301] higher FVC than those in the least active quartile (< 17 or < 21 min/day for females and males, respectively); however these associations were stronger among ex−/current smokers. Achieving at least once 10 consecutive minutes of MVPA was only associated with higher PI_max_ [β-estimate: 0.57 kPa; 95% CI: 0.04, 1.10], remaining significant among never smokers. No associations were found with diffusion capacity or for reaching the WHO-recommended 150 min of MVPA/week in 10-min bouts.

**Conclusions:**

Although the effects were small, active subjects showed higher spirometric results. The observed associations were more pronounced among ever smokers suggesting a higher benefit of PA for subjects being at a higher risk for chronic lung diseases.

**Electronic supplementary material:**

The online version of this article (10.1186/s12890-017-0562-8) contains supplementary material, which is available to authorized users.

## Background

Physical activity (PA) reduces the risk of premature mortality and chronic diseases like cardiovascular disease or diabetes mellitus [1]. Benefits of activity apply also to persons with chronic lung diseases such as asthma or chronic obstructive pulmonary disease (COPD), who are therefore encouraged to engage in regular PA [2, 3]. Studies have shown that higher PA was associated with a lower risk of hospital admissions and all-cause mortality in COPD patients [3, 4]. Furthermore, the diffusion capacity of the lung for carbon monoxide was shown to be a predictor of a decline in 6-min-walking distance in COPD patients [5]. Moreover, exercise training in COPD patients was associated with improved ventilatory muscle function and showed positive effects on the forced vital capacity (FVC) [6, 7]. Due to the positive health effects of PA in patients with chronic lung diseases, PA has been incorporated into pulmonary rehabilitation programs [8].

In population-based studies, PA was shown to be associated with slower age-related decline of the forced expiratory volume in 1 s (FEV_1_) in adults [9, 10]. Results from a longitudinal study among middle-aged men showed that those with higher levels of PA experienced slower lung function decline over 25 years [11]. However, all these studies assessed PA by questionnaires, which was found to correlate only low-to-moderately with activity objectively assessed by motion sensors in adults [12, 13].

Only a few studies have investigated the association of accelerometer-based PA with lung function. Moreover, the association between PA and lung function in lung-healthy persons is unclear. In lung-healthy adolescents, no associations were found between accelerometer-based PA and a broad range of spirometric parameters [14]. In adults, results of a study among 62 smokers showed that lung function between inactive participants, defined as those who engaged in less than 150 min/week of moderate to vigorous PA (MVPA), and active ones did not differ [15]. Thus, the evidence is inconclusive.

Furthermore, studies among athletes suggest that endurance exercise is associated with higher FVC and improved lung diffusion capacity [16, 17]. A study among 25 healthy men showed an improvement of maximal inspiratory pressure after 5 weeks of inspiratory muscle training [18], as observed in COPD patients after exercise training [6], whereas the increase in respiratory muscle endurance of marathon runners was described as a consequence of differences in breathing pattern developed during running rather than respiratory muscle strength [19].

Considering the limited evidence on the association between PA and lung function in lung-healthy populations, our aim was to investigate the association of accelerometer-based PA with lung function in apparently lung-healthy German adults from a population-based sample. Therefore, we addressed different aspects of lung function i.e. lung volume, airflow limitation, pulmonary gas exchange (TLCO/VA) and inspiratory muscle strength (PI_max_).

## Methods

### Study population

The present analysis was based on a follow-up study of the KORA S4 (KORA: Cooperative Health Research in the Region of Augsburg) cohort comprising 4261 adults (51.0% female) examined in 1999 – 2001. A description of the primary study design has been published previously [20].

**Fig. 1:**
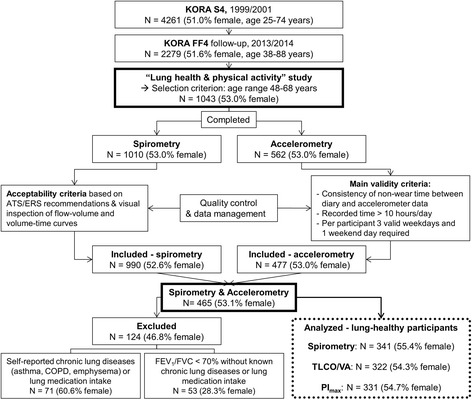
Selection of participants. KORA: Cooperative Health Research in the Region of Augsburg. ATS: American Thoracic Society. ERS: European Respiratory Society. COPD: chronic obstructive pulmonary disease. FEV_1_: forced expiratory volume in 1 s. FVC: forced vital capacity. TLCO/VA: transfer factor of the lung for carbon monoxide adjusted for hemoglobin and divided by alveolar volume. PI_max_: maximum inspiratory mouth pressure

The selection of the study population is displayed in detail in Fig. 1. In the KORA FF4 follow-up study 2279 participants (51.6% female) aged 38-88 years were examined between June 2013 and September 2014. Covering only the age range 48-68 years, 1043 of these participants were selected for the “Lung health & physical activity” study, which comprised spirometry, assessment of inspiratory muscle strength (PI_max_), measures of pulmonary gas exchange, and accelerometer-based assessment of habitual PA over one week. Information on sociodemographic variables, and current medication intake seven days before examination was obtained from standardized interviews and questionnaires. For the assessment of common diseases such as stroke or myocardial infarction, subjects were asked for each disease separately if a doctor has ever diagnosed this particular disease. Further, information on mobility and pain/discomfort was obtained from the EuroQol five dimensions questionnaire (EQ-5D 5 L) [21].

Of 1010 subjects who participated in spirometry, spirometric data of 990 participants were considered as valid based on international recommendations [22]. In accelerometry, two males with values greater than the mean plus seven times the standard deviation for weight and MVPA, respectively, were excluded during data management after quality control, resulting in 477 subjects with valid accelerometric data out of 562 who initially participated. For the selection of lung-healthy subjects, 465 subjects who had both, valid spirometry and accelerometry were considered. Subjects who reported a doctor’s diagnosis of asthma, emphysema, chronic bronchitis or COPD, or used pulmonary medication including antileukotrienes, inhaled sympathomimetics, anticholinergics, and/or steroids were excluded from this analysis (*N* = 71). Furthermore, subjects with FEV_1_/FVC < 0.7 (indication of airflow limitation) were excluded (*N* = 53) [23]. Finally, 341 apparently lung-healthy subjects were available for the present analysis. Among these, PI_max_ results were available for 331 subjects (54.7% female) and results for the transfer factor of the lung for carbon monoxide divided by alveolar volume (TLCO/VA) for 322 subjects (54.3% female).

### Physical activity assessment

A detailed description of the procedure, data handling, quality control and inclusion criteria has been reported previously [24]. In brief, participants were asked to wear an ActiGraph GT3X accelerometer (Pensacola, Florida) over a one week period at the hip from getting up until going to bed time and to complete a daily diary which included the time of getting up, going to sleep, and reasons for and duration of non-wear time. Non-wear time according to the accelerometer data was assessed based on the algorithm derived from the National Health and Nutrition Examination Survey [25]. Days were considered as not valid, if the difference between the non-wear time algorithm applied to the accelerometer data and the diary non-wear time was greater than 60 min (if the non-wear time was reported in the diary) or greater than 120 min (if the accelerometer indicated a non-wear time). Subjects were excluded in case of no reported non-wear time over the whole 7 day reporting period although the accelerometer should have been removed during water activities e.g. showering. Further exclusion criteria for single days were missing information on time spent awake, day length < 10 h/day, non-wear time during sport activities lasting > 2 h, or incorrect handling of the accelerometer e.g. hand instead of hip. Further details of all exclusion criteria have been previously published [24]. Subjects were only included in the analysis if they had at least 3 valid weekdays and 1 valid weekend day. 89% of the subjects included in our analysis recorded at least 6 days, 9% had 5 days and 2% provided 4 days only. Measured accelerations of the vertical axis were stored at 1 Hz and converted into 1-min epochs classifying a moderate to vigorous PA (MVPA) level as ≥ 1952 counts per minute, as proposed by Freedson et al. [26]. Non-wear time during sport was imputed as described here [14, 24].

Since the relationship between MVPA and lung function measures adjusted for sex, age, and height was non-linear, MVPA was divided into sex-specific quartiles. In addition, two further binary PA variables were built based on the World Health Organization (WHO) recommendation stating that adults should accumulate at least 150 min of MVPA/week in bouts of at least 10 min [27].

PA was quantified as exposure in three ways: (1) sex-specific MVPA quartiles; (2) achieving at least one 10-min bout of MVPA over the whole measurement period (yes vs. no); (3) reaching the WHO threshold of 150 min MVPA/week in at least 10-min bouts (yes vs. no). Cut-offs for sex-specific MVPA quartiles were set as follows: cut-off for males: 1st ≤ 21.6 min/day, 2nd > 21.6-35.2 min/day, 3rd > 35.2-49.9 min/day, 4th > 49.9 min/day; and cut-offs for females: 1st ≤ 17.2 min/day, 2nd > 17.2-28.3 min/day, 3rd > 28.3-46.7 min/day, 4th > 46.7 min/day, respectively.

### Lung function assessment

Lung function assessment was performed in line with the American Thoracic Society (ATS) and European Respiratory Society (ERS) statements [22, 28, 29].

Flow-volume curves were obtained using a pneumotachograph-type spirometer (MasterScope, Jaeger, Hoechberg, Germany). Subjects were guided to perform at least 3 and up to 8 spirometric maneuvers per test in order to obtain acceptable and reproducible values. During the maneuvers both flow-volume and volume-time curves were monitored online by a trained examiner. After each test, the curves were visually inspected, artifacts e.g. coughing were excluded and the results were selected and evaluated according to the ATS/ERS recommendations [22], including a good start with extrapolated volume < 0.5% of FVC or 0.15 l, an exhalation of ≥ 6 s or a plateau in volume-time curve. Spirometric parameters included FEV_1_, FVC, FEV_1_/FVC, and forced expiratory flow between 25% and 75% of exhaled FVC (FEF_25-75_). Standardized z-scores were calculated using reference equations for spirometry from the Global Lung Function Initiative (GLI) [30]. TLCO was determined using the single-breath technique. Subjects were asked to perform a maximum of 5 trials in order to achieve acceptable and reproducible values with an inspired volume > 85% of the largest vital capacity in <4 s, and an effective breath hold time within 8 to 12 s according to ATS/ERS recommendations [28]. TLCO results were adjusted for hemoglobin obtained from blood samples collected on the day of the physical examination in the study center [28]. For determination of PI_max_, subjects were instructed to exhale to residual volume followed by a maximal voluntary inspiration against an obstructed mouth piece with a small leak to prevent glottic closure using a flanged mouth piece [29]. The highest peak inspiratory pressure achieved during a minimum of 3 and a maximum of 15 maneuvers was used for analysis (MasterScreen PFT, Jaeger, Hoechberg, Germany).

### Statistical analyses

Sex-specific differences were assessed using Pearson’s Chi-squared test (categorical variables), the t-test (normal distribution), and Wilcoxon rank-sum test (skewed distribution). Mean and corresponding standard deviation or percentages (%, N) were used to describe subject characteristics and categorized PA. Due to a non-normal distribution, median and 1st and 3rd quartiles were reported for continuous MVPA.

Adjusted linear regression models were applied to analyze associations between PA and lung function parameters. Since spirometric parameters (FEV_1_, FVC, and FEF_25-75_) were correlated (*r* = 0.61 to 0.98), the results of regression analyses were not adjusted for multiple testing. For each spirometric parameter, the mean plus/minus 4 times the standard deviation was calculated for each sex to determine sex-specific outliers. According to this definition one subject was excluded in the analyses using z-scores for FEV_1_ and FVC and another in FEF_25-75_ models. The main model was adjusted for sex, age, height, weight, smoking status categorized as *never*, *ex-*, or *current* smokers, education level categorized as low (<10 years of school, i. e. “Hauptschule” in Germany), middle (10 years of school, i.e. “Realschule”) and high (>10 years of school, i.e. “Gymnasium”), and a doctor’s diagnosis of hay fever (ever). Regression models for standardized GLI z-scores [30] that are already adjusted for ethnicity, sex, age, and height, were adjusted only for additional variables. Covariates remained in the model independent of statistical significance. As the mean body mass index (BMI) was 27.7 kg/m^2^ we included a sensitivity analysis, replacing weight with BMI in the main analysis.

To assess if the association might be modified through other covariates potentially associated with lung function, sensitivity analyses were done. Since smoking behavior has an impact on lung function and might modify potential associations, we calculated the main regression model with stratification into never and ex−/current smokers. In further analyses, the main model was additionally adjusted for moderate to extreme problems in walking about and/or pain or discomfort, season categorized as winter (start of measurement: December to February), spring (March to May), summer (June to August), and autumn (September to November), or for self-reported acute respiratory infections in the last three weeks prior to lung function testing. All participants were Caucasian therefore ethnicity was not included as a covariate. Interaction effects between MVPA and sex were tested in the main model. In case of significant interaction effects (*p* < 0.05), stratified results were reported additionally.

To eliminate a possible impact of myocardial infarction and/or stroke (*N* = 15), subjects with a history of these events were excluded from the main model as a further sensitivity analysis.

The statistical program R, version 3.3.3 [31], was used for all analyses and *p*-values below 0.05 were considered statistically significant.

## Results

The study population consisted of 341 (45% male) apparently lung-healthy subjects (i.e. no chronic lung diseases or pulmonary medication intake, and FEV_1_/FVC ≥ 0.7) with a mean age of 57 years (Table 1). The prevalence of current smoking was 14%, while 47% of the participants reported to be never smokers. Despite a lower prevalence of females among all ex−/current smokers than among never smokers (50.0% and 61.6%, respectively), 70.2% of current smokers were female. BMI was comparable between never and ex−/current smokers. Mean z-scores were lower among ex−/current smokers compared to never smokers, being statistically significant for z-score FEF_25-75_. Nevertheless, smoking status did not affect PA (Additional file 1: Table S1). Included subjects were slightly younger (mean age of 57 years vs. 58 years, respectively) and more often never smokers (46.6% vs. 37.8%, respectively) compared to all other subjects performing spirometry (Additional file 1: Table S2).

**Table 1:** Population characteristics, lung function and physical activity measurements

	Males (*n* = 152)	Females (*n* = 189)
Age		
mean years (SD)	57.1 (5.8)	57.5 (5.3)
Height *		
mean cm (SD)	177.0 (6.0)	162.1 (6.0)
Weight *		
mean kg (SD)	88.8 (13.5)	71.5 (14.4)
BMI*		
n normal (BMI < 25) (%)	27 (17.8)	79 (41.8)
n overweight (≥ 25 BMI <30) (%)	83 (54.6)	61 (32.3)
n obese (BMI ≥ 30) (%)	42 (27.6)	49 (25.9)
Smoking status*		
n never smokers (%)	61 (40.1)	98 (51.9)
n ex-smokers (%)	77 (50.7)	58 (30.7)
n current smokers (%)	14 (9.2)	33 (17.5)
Education*		
n low (< 10 years of school) (%)	68 (44.7)	84 (44.4)
n medium (10 years of school) (%)	34 (22.4)	66 (34.9)
n high (> 10 years of school) (%)	50 (32.9)	39 (20.6)
Hay fever		
n yes (%)	22 (14.5)	43 (22.8)
Problems in walking about, and/or pain or discomfort		
n not at all/slight (%)	135 (88.8)	154 (81.9)
n moderate/extreme (%)	17 (11.2)	34 (18.1)
Lung function		
FEV_1_*		
mean l (SD)	3.76 (0.57)	2.66 (0.42)
FVC*		
mean l (SD)	4.88 (0.71)	3.39 (0.56)
FEV_1_/FVC*		
mean % (SD)	77.08 (3.93)	78.5 (4.14)
FEF_25-75_*		
mean l/s (SD)	3.16 (0.91)	2.37 (0.63)
Z-score FEV_1_		
mean (SD)	0.25 (0.86)	0.32 (0.91)
Z-score FVC		
mean (SD)	0.29 (0.80)	0.35 (0.87)
Z-score FEV_1_/FVC		
mean (SD)	−0.12 (0.58)	−0.15 (0.63)
Z-score FEF_25-75_		
mean (SD)	−0.01 (0.78)	−0.01 (0.80)
TLCO, hemoglobin adjusted*		
mean mmol/min/kPa (SD)	9.88 (1.50)	6.90 (1.07)
TLCO/VA*		
mean mmol/min/kPa/l (SD)	1.45 (0.16)	1.38 (0.18)
PI_max_*		
mean kPa (SD)	9.77 (2.53)	6.67 (2.12)
Physical activity		
MVPA*		
median of mean min/day (1st; 3rd quartile)	35.2 (21.6; 49.9)	28.3 (17.2; 46.7)
10-min bout of MVPA achieved		
n yes (%)	104 (68.4)	120 (63.5)
WHO threshold achieved		
n yes (%)	24 (15.8)	26 (13.8)

*SD* standard deviation, *BMI* body mass index, *FEV*_*1*_ forced expiratory volume in 1 s, *FVC* forced vital capacity, *FEF*_*25-75*_ forced expiratory flow between 25% and 75% of FVC, *TLCO/VA* transfer factor of the lung for carbon monoxide adjusted for hemoglobin and divided by alveolar volume, *PI*_*max*_ maximum inspiratory mouth pressure, *MVPA* moderate to vigorous physical activity

*p < 0.05 (males vs. females)

Overall, participants spent a median of 31 min/day in MVPA with a range from 1 to 111 mean min/day, being lower for females than for males (median 28 vs. 35 min, respectively). In total, 66% of subjects achieved at least one 10-min bout of MVPA, and 15% achieved the recommended 150 min of MVPA/week in at least 10-min bouts (Table 1).

### Physical activity and spirometric parameters

In the total population, being in the most active MVPA quartile was associated with higher FEV_1_, FVC, FEV_1_ z-score and FVC z-score (Table 2, Additional file 1: Table S3). FEV_1_ was 142 ml higher in the most active subjects, i.e. females that engaged > 47 min/day and males that engaged > 50 min/day in MVPA, than in the least active quartile, i.e. < 17 min/day for females or < 21 min/day for males. Stratified analyses revealed that these associations remained in ex−/current smokers, but not in never smokers (Table 2).

**Table 2:** Association of physical activity with spirometric parameters

	MVPA	Total population (*n* = 341)		Never smokers (*n* = 159)		Ex- and current smokers (*n* = 182)	
	Quartile^a^	β (95% CI)	*p*-value	β (95% CI)	*p*-value	β (95% CI)	*p*-value
FEV_1_, ml	2	72 (−44, 188)	0.22	44 (−129, 218)	0.62	81 (−78, 240)	0.32
	3	**124 (7, 242)**	**0.04**	167 (−7, 341)	0.06	60 (−104, 224)	0.47
	4	**142 (23, 260)**	**0.02**	84 (−89, 257)	0.34	**195 (29, 360)**	**0.02**
FVC, ml	2	91 (−52, 235)	0.21	48 (−160, 256)	0.65	114 (−87, 316)	0.27
	3	107 (−38, 251)	0.15	190 (−18, 398)	0.08	11 (−196, 219)	0.92
	4	**155 (10, 301)**	**0.04**	75 (−132, 282)	0.48	**227 (18, 437)**	**0.04**
FEV_1_/FVC, %	2	0.01 (−1.18, 1.21)	0.98	−0.03 (−1.86, 1.80)	0.97	−0.10 (−1.72, 1.51)	0.90
	3	0.87 (−0.34, 2.07)	0.16	0.33 (−1.50, 2.17)	0.72	1.07 (−0.59, 2.74)	0.21
	4	0.64 (−0.57, 1.85)	0.30	0.70 (−1.12, 2.52)	0.45	0.56 (−1.12, 2.24)	0.51
FEF_25-75_, ml/s	2	100 (−120, 319)	0.37	61 (−266, 388)	0.71	101 (−201, 403)	0.51
	3	**277 (56, 499)**	**0.01**	239 (−89, 566)	0.16	246 (−65, 558)	0.12
	4	161 (−63, 384)	0.16	106 (−220, 433)	0.52	213 (−102, 527)	0.19

*MVPA* moderate to vigorous physical activity, *CI* confidence interval, *FEV*_*1*_ forced expiratory volume in 1 s, *FVC* forced vital capacity, *FEF*_*25-75*_ forced expiratory flow between 25% and 75% of FVC

^a^Least active MVPA quartile (1st quartile) was used as reference. All models were adjusted for sex, age, height, weight, education level, a doctor’s diagnosis of hay fever and depending on the population analyzed also by smoking status (never, ex- or current)

Statistically significant associations (p < 0.05) are shown in bold

The results for FVC were comparable to those observed for FEV_1_, except for an interaction effect between the third MVPA quartile and sex that was present for FVC only. After stratification an association was found for females only (Additional file 1: Table S4). Sensitivity analyses, e.g. further adjustment for walking difficulties and/or discomfort, and exclusion of subjects with stroke and/or myocardial infarction did not substantially change our results. Adjusting for BMI instead of weight led to the very similar results (Additional file 1: Table S5).

When MVPA was quantified as achieving at least one 10-min bout, an association with lung function was found in sex-stratified analyses for FEV_1_, FVC, z-scores for FEV_1_ and FVC in females only (Additional file 1: Tables S4 and S6). Reaching the recommended 150 min of MVPA/week in at least 10-min bouts was negatively associated with FEV_1_, FVC, and z-scores for FEV_1_ and FVC in males only (Additional file 1: Tables S4 and S7).

### Physical activity and inspiratory muscle strength

Subjects who engaged in at least one 10-min bout of MVPA had an estimated increase of PI_max_ by 0.6 kPA (Table 3). However, in analyses stratified by smoking status, this association was significant only in never smokers (Additional file 1: Table S8).

**Table 3:** Association of physical activity with pulmonary gas exchange and inspiratory muscle strength

	Pulmonary gas exchange (TLCO/VA), 10^−1^ mmol/min/kPa/l		Maximum inspiratory mouth pressure (PI_max_), kPa	
	β (95% CI)	*p*-value	β (95% CI)	*p*-value
MVPA quartiles,				
1st – least active (reference)	–	–	–	–
2nd	0.01 (−0.47, 0.49)	0.98	0.39 (−0.32, 1.10)	0.28
3rd	−0.30 (−0.80, 0.19)	0.23	0.23 (−0.48, 0.94)	0.53
4th – most active	0.25 (−0.25, 0.75)	0.33	0.05 (−0.68, 0.78)	0.90
10-min bout of MVPA achieved,				
yes vs. no	−0.03 (−0.39, 0.34)	0.88	**0.57 (0.04, 1.10)**	**0.04**
WHO threshold achieved,				
yes vs. no	0.36 (−0.13, 0.84)	0.15	0.00 (−0.71, 0.71)	1.00

The models were adjusted for sex, age, height, weight, smoking status categorized as *never*, *ex-*, or *current* smokers, education level, and a doctor’s diagnosis of hay fever

*TLCO/VA* transfer factor of the lung for carbon monoxide adjusted for hemoglobin and divided by alveolar volume, *PI*_*max*_ maximum inspiratory mouth pressure, *CI* confidence interval, *MVPA* moderate to vigorous physical activity

### Physical activity and pulmonary gas exchange

PA was not associated with TLCO/VA in any analysis, except for a negative association found with the third MVPA quartile among never smokers (Table 3, Additional file 1: Table S8).

## Discussion

The present analyses revealed weak positive associations between the most active subjects and volumetric indices in adults without lung function limitation. These associations were primarily observed among ex−/current smokers, but not in never smokers, suggesting that the effect might be driven by smoking behaviour. While PA showed no association with TLCO/VA, PI_max_ was higher in subjects who engaged in at least one 10-min bout of MVPA during the recording period, compared to those who did not.

Compared to current recommendations PA was rather low in our population-based cohort. Median daily MVPA was 31 min, and only 15% of subjects met the WHO PA recommendation. This finding, however, is comparable to results obtained through the questionnaire-based German Health Interview and Examination Survey for Adults where 18% of 50-69 year old participants achieved the WHO activity threshold [32].

Still, weak associations with lung function were present in our analyses.

An association between PI_max_ and the achievement of at least one 10-min bout of MVPA was found in our population, remaining significant in never smokers only. In COPD, twitch mouth pressure has previously been shown to decrease with increasing disease severity and PI_max_ could be improved by exercise training [6, 33]. A study among marathon athletes compared to sedentary controls reported a higher respiratory muscle endurance in the athletes - probably as a result of breath technique, but a similar PI_max_ [19].

In our population, those who reached at least one 10-min bout spent in MVPA showed a higher PI_max_, while reaching the WHO PA recommendation showed no increase. It might be that the threshold of engaging for at least 10 consecutive minutes in habitual MVPA, i.e. separating subjects with short bouts of activity and those with at least sporadic activity for 10 min, might represent a plateau. However, this result should be interpreted with caution due to the weak associations found and the potentially minor clinical relevance.

While TLCO has previously been shown to be a clinically relevant predictor of exercise capacity determined by 6-min-walking distance test in COPD patients [5], PA was not found to be a predictor of TLCO/VA in our apparently lung-healthy population. The lack of findings might be due to the possibly small effects that could not be detected in habitual PA of middle-aged adults without lung function limitation.

Our results show a weak, but positive association of PA and volumetric lung function indices, primarily seen among ex−/current smokers. Compared to the least active subjects, subjects engaging on average more than about 48 min/day in MVPA had a 142 ml higher FEV_1_. Considering an annual decline of around 25 ml of FEV_1_ in adults [34, 35], our results would correspond to an age-related decline of about 5 years. No causal relationships or long-term effects can be drawn from our cross-sectional analysis, but our results are in line with those from a longitudinal study observing that current smokers with moderate and high PA had a decreased decline in FEV_1_ and FVC compared with smokers with low PA [36]; and as in our study, this association was not observed in never smokers. A study including only smokers did not find an association between the achievement of at least 150 min/week of MVPA and spirometric parameters [15]. However, observed differences may be related to diverse designs, population characteristics and definitions of being active [15, 36]. PA was suggested to promote an anti-inflammatory status and to potentially protect against chronic diseases associated with low-grade systemic inflammation [37]. Smokers are at a higher risk for COPD and other smoking-related diseases typically experiencing a low-grade systemic inflammation [38]. In our analysis, the positive associations between being active and volumetric indices were more pronounced among ex−/current smokers, suggesting a higher benefit of PA for subjects being at a higher risk for chronic lung diseases.

### Strength and limitations

A major strength of this study is the investigation of a range of standardized lung function parameters obtained by spirometry, as well as less often investigated measures of gas exchange and respiratory muscle strength in apparently lung-healthy adults. We objectively assessed PA by accelerometry, which is rare, as most available studies assessed PA by questionnaires [9–11, 36]. Evidence for the association between PA and lung function in the general population without chronic lung diseases is likewise rare.

Due to the cross-sectional design of our analysis, it is not possible to draw conclusions about long-term effects or causal relations. Although associations were found, these results should be interpreted with caution due to the small effects seen with partly wide confidence intervals. Further, the present results are limited to the pre-selected lung-healthy study population, comprising 48-68 year old residents in the region of Augsburg in southern Germany. Information on chronic lung diseases was assessed via self-report and was not individually verified by a physician.

## Conclusions

Objective measurements of physical activity showed a weak, but positive association with slightly higher volumetric lung function indices in lung-healthy adults from southern Germany. This association was mainly observed among ex−/current smokers. Further, engaging in MVPA for at least 10 consecutive minutes was associated with higher PI_max_, remaining significant in never smokers only. No associations were found for TLCO/VA. Although the effects were small, our results suggest a positive association of PA with lung function of lung-healthy subjects from a population-based cohort.

## Additional file

Additional file 1: Results of Tables S1 to S8. (PDF 521 kb)

## Abbreviations

BMI: Body mass index
CI: Confidence interval
FEF_25-75_: Forced expiratory flow between 25% and 75% of FVC
FEV_1_: Forced expiratory volume in 1 s
FVC: Forced vital capacity
GLI: Global Lung Function Initiative
MVPA: Moderate to vigorous physical activity
PA: Physical activity
PI_max_: Maximum inspiratory mouth pressure
SD: Standard deviation
TLCO/VA: Transfer factor of the lung for carbon monoxide adjusted for hemoglobin and divided by alveolar volume
WHO: World Health Organization
