# Supplementary material for: Association of physical activity with lung function in lung-healthy German adults: results from the KORA FF4 study
Source: BMC Pulm Med. 2017 Dec 28;17:215. doi: 10.1186/s12890-017-0562-8 (PMC5745968; doi:10.1186/s12890-017-0562-8)
Supplement: Additional file 1: — Results of Tables S1 to S8. (PDF 521 kb) [file 12890_2017_562_MOESM1_ESM.pdf]

Additional file 1: Results of Tables S1 to S8

Table S1 Population characteristics stratified by smoking status

		Never smokers (n=159)	Ex- and current smokers (n=182)
**Sex***	n females (%)	98 (61.6)	91 (50.0)
**Age**	mean years (SD)	57.6 (5.5)	57.2 (5.5)
**Height***	mean cm (SD)	167.5 (9.7)	169.9 (9.3)
**Weight***	mean kg (SD)	77.3 (16.6)	80.9 (16.2)
**BMI**	n normal (BMI <25 BMI) (%)	54 (34.0)	52 (28.6)
	n overweight (≥25 BMI <30) (%)	68 (42.8)	76 (41.8)
	n obese (BMI ≥30) (%)	37 (23.3)	54 (29.7)
**Education**	n low (<10 years of school) (%)	62 (39.0)	90 (49.5)
	n medium (=10 years of school) (%)	54 (34.0)	46 (25.3)
	n high (>10 years of school) (%)	43 (27.0)	46 (25.3)
**Hay fever**	n yes (%)	33 (20.8)	32 (17.6)
**Problems in walking about/pain/discomfort**	n not at all/slight (%)	133 (84.2)	156 (85.7)
	n moderate/extreme (%)	25 (15.8)	26 (14.3)
**Lung function**			
**Z-score FEV_1**	mean (SD)	0.34 (0.92)	0.24 (0.86)
**Z-score FVC**	mean (SD)	0.33 (0.84)	0.32 (0.85)
**Z-score FEV_1/FVC**	mean (SD)	-0.07 (0.61)	-0.20 (0.60)
**Z-score FEF_{25-75}***	mean (SD)	0.08 (0.82)	-0.09 (0.76)
**TLCO/VA***	mean mmol/min/kPa/l (SD)	14.39 (1.68)	13.85 (1.82)
**PI_{max}***	mean kPa (SD)	7.75 (2.86)	8.36 (2.68)
**Physical activity**			
**MVPA among females**	median of mean minutes/day (1st; 3rd quartile)	28.7 (17.2; 49.6)	28.0 (17.5; 46.3)
**MVPA among males**	median of mean minutes/day (1st; 3rd quartile)	36.6 (23.1; 52.0)	33.0 (21.0; 48.7)
**MVPA quartiles**	n 1st – least active (%)	38 (23.9)	47 (25.8)
	n 2nd (%)	37 (23.3)	48 (26.4)
	n 3rd (%)	41 (25.8)	44 (24.2)
	n 4th – most active (%)	43 (27.0)	43 (23.6)
**10-minute bout of MVPA achieved**	n yes (%)	109 (68.6)	115 (63.2)
**WHO threshold achieved**	n yes (%)	26 (16.4)	24 (13.2)

*p<0.05 between never and ex-/current smokers.

SD: standard deviation. BMI: body mass index. FEV_1: forced expiratory volume in 1 second. FVC: forced vital capacity. FEF_{25-75}: forced expiratory flow between 25% and 75% of FVC. TLCO/VA: transfer factor of the lung for carbon monoxide adjusted for hemoglobin and divided by alveolar volume. PI_{max}: maximum inspiratory mouth pressure. MVPA: moderate to vigorous physical activity. WHO: World Health Organization.

Table S2 Comparison of subjects included in the present analysis compared to all other participants of the "Lung Health & physical activity" study performing spirometry

		**Analyzed population (n=341)**	**"Lung Health & physical activity" study participants with spirometry (n=669)**
Sex	n female (%)	189 (55.4)	346 (51.7)
Age*	mean years (SD)	57.4 (5.5)	58.4 (5.8)
Height	mean cm (SD)	168.7 (9.6)	169.3 (9.3)
Weight	mean kg (SD)	79.2 (16.4)	81.0 (18.4)
BMI	n normal (BMI <25 BMI) (%)	106 (31.1)	194 (29.0)
	n overweight (≥25 BMI <30) (%)	144 (42.2)	273 (40.8)
	n obese (BMI ≥30) (%)	91 (26.7)	202 (30.2)
Smoking status*	n never smokers (%)	159 (46.6)	253 (37.8)
	n ex-smokers (%)	135 (39.6)	280 (41.9)
	n current smokers (%)	47 (13.8)	136 (20.3)
Education	n low (<10 years of school) (%)	152 (44.6)	325 (48.8)
	n medium (=10 years of school) (%)	100 (29.3)	164 (24.6)
	n high (>10 years of school) (%)	89 (26.1)	177 (26.6)
Hay fever	n yes (%)	65 (19.1)	140 (20.9)
Asthma	n yes (%)	-	98 (14.6)
COPD or emphysema	n yes (%)	-	83 (12.4)
Problems in walking about/pain/discomfort*	n not at all/slight (%)	289 (85.0)	530 (79.3)
	n moderate/extreme (%)	51 (15.0)	138 (20.7)

*p<0.05 between study population analyzed and all other subjects participating in spirometry.
SD: standard deviation. BMI: body mass index. COPD: chronic obstructive pulmonary disease.

Table S3 Association of physical activity with GLI z-scores for spirometric parameters

	MVPA	Total population[a] (n=341)		Never smokers (n=159)		Ex- and current smokers[b] (n=182)	
	Quartile	β (95% CI)	p-value	β (95% CI)	p-value	β (95% CI)	p-value
Z-score FEV_1	2	0.17 (-0.10, 0.43)	0.21	0.08 (-0.34, 0.49)	0.72	0.26 (-0.08, 0.60)	0.14
	3	**0.27 (0.01, 0.53)**	**0.05**	0.35 (-0.06, 0.76)	0.10	0.20 (-0.15, 0.55)	0.26
	4	**0.34 (0.07, 0.60)**	**0.01**	0.20 (-0.20, 0.60)	0.33	**0.48 (0.12, 0.83)**	**0.01**
Z-score FVC	2	0.18 (-0.06, 0.43)	0.14	0.10 (-0.27, 0.47)	0.60	0.27 (-0.06, 0.60)	0.11
	3	0.21 (-0.04, 0.46)	0.10	0.34 (-0.02, 0.71)	0.07	0.09 (-0.24, 0.43)	0.59
	4	**0.31 (0.06, 0.56)**	**0.01**	0.18 (-0.18, 0.54)	0.33	**0.45 (0.10, 0.80)**	**0.01**
Z-score FEV_1/FVC	2	-0.04 (-0.22, 0.14)	0.65	-0.04 (-0.32, 0.23)	0.76	-0.04 (-0.27, 0.20)	0.77
	3	0.09 (-0.09, 0.26)	0.35	-0.01 (-0.29, 0.26)	0.93	0.17 (-0.07, 0.41)	0.18
	4	0.04 (-0.14, 0.22)	0.63	0.03 (-0.24, 0.30)	0.85	0.06 (-0.19, 0.31)	0.64
Z-score FEF_{25-75}	2	0.08 (-0.16, 0.31)	0.52	-0.01 (-0.38, 0.37)	0.97	0.16 (-0.14, 0.46)	0.31
	3	**0.25 (0.01, 0.48)**	**0.04**	0.21 (-0.16, 0.57)	0.27	0.27 (-0.04, 0.58)	0.09
	4	0.18 (-0.06, 0.41)	0.15	0.01 (-0.27, 0.46)	0.61	0.26 (-0.06, 0.58)	0.11

All models were adjusted for weight, education level, and a doctor's diagnosis of hay fever.

[a]additionally adjusted for smoking status categorized as *never*, *ex-*, or *current* smokers.

[b]additionally adjusted for smoking status categorized as *ex-* or *current* smokers.

GLI: Global Lung Function Initiative. MVPA: moderate to vigorous physical activity. CI: confidence interval. FEV_1: forced expiratory volume in 1 second. FVC: forced vital capacity. FEF_{25-75}: forced expiratory flow between 25% and 75% of FVC.

Table S4 Sex-stratified results of linear regression models

		Males (n=152)		Females (n=189)	
		β (95% CI)	p-value	β (95% CI)	p-value
**MVPA**	Quartile				
FEV_1, ml	2	44 (-160, 248)	0.68	113 (-22, 247)	0.10
	3	45 (-158, 248)	0.66	**202 (66, 339)**	**0.00**
	4	60 (-148, 268)	0.57	**209 (73, 345)**	**0.00**
FVC, ml	2	52 (-192, 297)	0.67	144 (-26, 314)	0.10
	3	-72 (-315,171)	0.56	**255 (83, 428)**	**0.00**
	4	88 (-161, 337)	0.49	**210 (38, 381)**	**0.02**
FEV_1/FVC, %	2	-0.10 (-1.81, 1.6)	0.90	0.16 (-1.48, 1.80)	0.85
	3	**2.04 (0.34, 3.75)**	**0.02**	0.15 (-1.52, 1.82)	0.86
	4	-0.21 (-1.96, 1.53)	0.81	1.35 (-0.31, 3.01)	0.11
Z-score FEV_1/FVC	2	-0.02 (-0.27, 0.24)	0.90	-0.04 (-0.28, 0.21)	0.77
	3	**0.31 (0.05, 0.56)**	**0.02**	-0.05 (-0.30, 0.20)	0.69
	4	-0.05 (-0.3, 0.21)	0.73	0.17 (-0.08, 0.41)	0.20
**10-minute bout of MVPA achieved**, yes vs. no					
FEV_1, ml		-70 (-229, 90)	0.39	**154 (49, 259)**	**0.00**
FVC, ml		-121 (-312, 70)	0.22	**157 (23, 290)**	**0.02**
Z-score FEV_1		-0.11 (-0.42, 0.20)	0.48	**0.36 (0.08, 0.64)**	**0.01**
Z-score FVC		-0.15 (-0.43, 0.14)	0.31	**0.30 (0.04, 0.57)**	**0.03**
**WHO threshold achieved**, yes vs. no					
FEV_1, ml		**-196 (-387, -5)**	**0.05**	105 (-39, 249)	0.15
FVC, ml		**-242 (-472, -12)**	**0.04**	79 (-103, 261)	0.39
Z-score FEV_1		**-0.38 (-0.75, 0.00)**	**0.05**	0.33 (-0.05, 0.71)	0.09
Z-score FVC		**-0.35 (-0.70, -0.01)**	**0.05**	0.25 (-0.12, 0.61)	0.18

The models were adjusted for sex, age, height, weight, smoking status, education level, and a doctor's diagnosis of hay fever.

MVPA: moderate to vigorous physical activity. CI: confidence interval. FEV_1: forced expiratory volume in 1 second. FVC: forced vital capacity. WHO: World Health Organization.

Table S5 Association of physical activity with spirometric parameters with **adjustment for BMI instead of weight**

	MVPA	Total population[a] (n=341)		Never smokers (n=159)		Ex- and current smokers[b] (n=182)	
	Quartile	β (95% CI)	p-value	β (95% CI)	p-value	β (95% CI)	p-value
FEV_1, ml	2	71 (-45, 187)	0.23	43 (-131, 216)	0.63	81 (-79, 240)	0.32
	3	**124 (7, 242)**	**0.04**	163 (-11, 337)	0.07	61 (-103, 225)	0.47
	4	**141 (22, 259)**	**0.02**	79 (-94, 252)	0.37	**197 (31, 363)**	**0.02**
FVC, ml	2	89 (-54, 233)	0.22	45 (-162, 252)	0.67	113 (-89, 315)	0.27
	3	106 (-39, 251)	0.15	183 (-25, 391)	0.09	13 (-195, 222)	0.90
	4	**154 (8, 300)**	**0.04**	68 (-139, 275)	0.52	**231 (21, 441)**	**0.03**
FEV_1/FVC, %	2	0.03 (-1.16, 1.22)	0.96	-0.01 (-1.84, 1.82)	0.99	-0.09 (-1.71, 1.53)	0.91
	3	0.87 (-0.33, 2.07)	0.16	0.36 (-1.48, 2.19)	0.70	1.06 (-0.61, 2.73)	0.21
	4	0.65 (-0.56, 1.86)	0.29	0.73 (-1.09, 2.56)	0.43	0.55 (-1.13, 2.23)	0.52

All models were adjusted for sex, age, height, **body mass index**, education level, and a doctor's diagnosis of hay fever.

[a]additionally adjusted for smoking status categorized as *never*, *ex*-, or *current* smokers.

[b]additionally adjusted for smoking status categorized as *ex*- or *current* smokers.

BMI: body mass index. MVPA: moderate to vigorous physical activity. CI: confidence interval. FEV_1: forced expiratory volume in 1 second. FVC: forced vital capacity. $FEF_{25\text{-}75}$: forced expiratory flow between 25% and 75% of FVC.

Table S6 Association of at least one 10-minute bout of MVPA achieved with spirometric parameters

	**10-minute bout of MVPA achieved**, yes vs. no					
	Total population[a] (n=341)		Never smokers (n=159)		Ex- and current smokers[b] (n=182)	
	β (95% CI)	p-value	β (95% CI)	p-value	β (95% CI)	p-value
**FEV_1, ml**	36 (-52, 125)	0.42	74 (-59, 207)	0.28	22 (-98, 141)	0.72
**FVC, ml**	27 (-82, 136)	0.62	44 (-116, 203)	0.59	33 (-118, 185)	0.67
**FEV_1/FVC, %**	0.39 (-0.51, 1.29)	0.40	1.19 (-0.18, 2.57)	0.09	-0.23 (-1.43, 0.98)	0.71
**FEF_{25-75}, ml/s**	49 (-118, 216)	0.57	157 (-92, 405)	0.22	-28 (-253, 197)	0.81
**Z-score FEV_1**	0.13 (-0.07, 0.33)	0.21	0.16 (-0.16, 0.47)	0.33	0.12 (-0.15, 0.38)	0.39
**Z-score FVC**	0.11 (-0.08, 0.30)	0.24	0.08 (-0.20, 0.36)	0.58	0.15 (-0.11, 0.41)	0.25
**Z-score FEV_1/FVC**	0.03 (-0.10, 0.17)	0.63	0.12 (-0.09, 0.33)	0.26	-0.04 (-0.22, 0.14)	0.64
**Z-score FEF_{25-75}**	0.08 (-0.10, 0.26)	0.38	0.17 (-0.11, 0.45)	0.23	-0.01 (-0.24, 0.22)	0.94

The models were adjusted for sex, age, height, weight, education level, and a doctor's diagnosis of hay fever.

[a]additionally adjusted for smoking status categorized as *never*, *ex-*, or *current* smokers.

[b]additionally adjusted for smoking status categorized as *ex-* or *current* smokers.

MVPA: moderate to vigorous physical activity. CI: confidence interval. FEV_1: forced expiratory volume in 1 second. FVC: forced vital capacity. FEF_{25-75}: forced expiratory flow between 25% and 75% of FVC.

Table S7 Association of the achievement of WHO threshold with spirometric parameters

	**WHO threshold achieved**, yes vs. no					
	Total population[a] (n=341)		Never smokers (n=159)		Ex- and current smokers[b] (n=182)	
	β (95% CI)	p-value	β (95% CI)	p-value	β (95% CI)	p-value
**FEV_1, ml**	-35 (-153, 82)	0.56	60 (-107, 226)	0.48	-142 (-312, 27)	0.10
**FVC, ml**	-69 (-214, 75)	0.35	56 (-143, 254)	0.58	**-220 (-434, -6)**	**0.05**
**FEV_1/FVC, %**	0.45 (-0.75, 1.65)	0.46	0.42 (-1.31, 2.15)	0.64	0.65 (-1.07, 2.37)	0.46
**FEF_{25-75}, ml/s**	-31 (-255, 193)	0.79	18 (-299, 335)	0.91	-62 (-383, 260)	0.71
**Z-score FEV_1**	0.01 (-0.25, 0.28)	0.93	0.24 (-0.15, 0.63)	0.23	-0.24 (-0.61, 0.13)	0.20
**Z-score FVC**	-0.01 (-0.26, 0.24)	0.91	0.21 (-0.14, 0.55)	0.24	-0.26 (-0.62, 0.10)	0.16
**Z-score FEV_1/FVC**	0.04 (-0.14, 0.22)	0.66	0.01 (-0.25, 0.27)	0.94	0.07 (-0.18, 0.33)	0.57
**Z-score FEF_{25-75}**	0.01 (-0.22, 0.25)	0.92	0.08 (-0.27, 0.43)	0.66	-0.06 (-0.39, 0.26)	0.70

The models were adjusted for sex, age, height, weight, education level, and a doctor's diagnosis of hay fever.

[a]additionally adjusted for smoking status categorized as *never*, *ex-*, or *current* smokers.

[b]additionally adjusted for smoking status categorized as *ex-* or *current* smokers.

WHO: World Health Organization. CI: confidence interval. FEV_1: forced expiratory volume in 1 second. FVC: forced vital capacity. FEF_{25-75}: forced expiratory flow between 25% and 75% of FVC.

Table S8 Association of physical activity with pulmonary gas exchange and inspiratory muscle strength according to smoking status

	**Pulmonary gas exchange (TLCO/VA)**, 10^{-1} mmol/min/kPa/l				**Maximum inspiratory mouth pressure (PI_{max})**, kPa			
	Never smokers (n=151)		Ex- and current smokers[a] (n=171)		Never smokers (n=155)		Ex- and current smokers[a] (n=176)	
	β (95% CI)	p-value	β (95% CI)	p-value	β (95% CI)	p-value	β (95% CI)	p-value
**MVPA quartiles[a]**								
1st – least active vs.								
2nd	-0.26 (-0.93, 0.42)	0.45	0.22 (-0.47, 0.91)	0.53	0.85 (-0.21, 1.92)	0.12	0.01 (-0.96, 0.98)	0.98
3rd	**-0.81 (-1.50, -0.13)**	**0.02**	0.12 (-0.61, 0.85)	0.75	0.84 (-0.22, 1.91)	0.12	-0.20 (-1.20, 0.80)	0.70
4th – most active	-0.05 (-0.73, 0.64)	0.90	0.47 (-0.27, 1.20)	0.21	0.17 (-0.90, 1.23)	0.76	-0.01 (-1.05, 1.02)	0.98
**10-minute bout of MVPA achieved**, yes vs. no	0.00 (-0.54, 0.53)	0.99	-0.09 (-0.61, 0.42)	0.72	**0.93 (0.11, 1.74)**	**0.03**	0.37 (-0.35, 1.09)	0.32
**WHO threshold achieved**, yes vs. no	0.12 (-0.55, 0.79)	0.72	0.56 (-0.16, 1.28)	0.13	0.50 (-0.51, 1.51)	0.33	-0.51 (-1.55, 0.53)	0.33

All models were adjusted for sex, age, height, weight, education level, and a doctor's diagnosis of hay fever.

[a]additionally adjusted for smoking status categorized as *ex-* or *current* smokers.

TLCO/VA: transfer factor of the lung for carbon monoxide adjusted for hemoglobin and divided by alveolar volume. PI_{max}: maximum inspiratory mouth pressure. CI: confidence interval. MVPA: moderate to vigorous physical activity. WHO: World Health Organization.
